# Correlation between the results of three physical fitness tests (endurance, strength, speed) and the output measured during a bicycle ergometer test in a cohort of military servicemen

**DOI:** 10.1186/s40779-016-0083-4

**Published:** 2016-04-23

**Authors:** Stefan Sammito, Nils Gundlach, Irina Böckelmann

**Affiliations:** Bundeswehr Medical Service Headquarters, Section Health Promotion, Sports and Nutrition Medicine, Koblenz, Germany; Otto von Guericke University Magdeburg, Medical Faculty, Department of Occupational Medicine, Magdeburg, Germany; Bundeswehr Medical Clinic Rotenburg, Rotenburg (Wümme), Germany

**Keywords:** Physical fitness, Military, Ergometer test, Bicycle, Treadmill

## Abstract

**Background:**

Physical fitness tests are widely used to assess endurance, sprint ability, coordination and/or strength. The objective of the present study was to analyze the degree to which the results of the Bundeswehr Basis Fitness Test (BFT)--a physical fitness test comprising a sprint test (11 × 10-m shuttle test), a flexed-arm hang test and a 1000-m run--are consistent with the output measured during a bicycle ergometer test. The number of false-positive and false-negative results with regard to the assessment of physical fitness were also examined.

**Methods:**

As part of a retrospective study, health assessments from 323 reenlistment examinations were evaluated regarding the output measured during a bicycle ergometer test and compared with the BFT results of the candidates.

**Results:**

Overall, a good correlation was shown between the bicycle ergometer test results and the results achieved in the BFT disciplines. All three disciplines of the BFT showed a highly significant correlation with the relative output achieved during the bicycle ergometer test (*P* < 0.001), and also, the overall BFT score was highly significantly correlated (*P* < 0.001). The overall rate of false-positive and false-negative results was 4.0 %.

**Conclusions:**

The BFT results measured in the three physical fitness test items were highly correlated with the output measured during the bicycle ergometer tests. The rate of false-positive and false-negative results was low. The test items thus represent an appropriate measurement instrument because the test items require few equipment and less time. Additionally, a large number of subjects can be assessed. We suggest that it would be more useful to assess the physical fitness of this special group exclusively on the basis of the BFT instead of using the bicycle ergometer test.

## Background

Physical fitness tests are widely used to assess endurance, sprint ability, coordination and/or strength. Sets of various performance tests are especially useful in areas where, outside of a competitive sports context, a number of these abilities are essential for the accomplishment of tasks. For years, the level of physical fitness of military applicants and active soldiers--who must have a generally high fitness level [1–3]--has been evaluated based on national military fitness tests, both in Anglo-American countries [4–8] and in Europe [9, 10]. Because of country-specific particularities, the tests comprise different items. In most cases, however, endurance, strength and speed are assessed.

In 2010, the Basic Fitness Test (BFT) became compulsory in the German Armed Forces to evaluate physical fitness outside of medical examinations [11]. Every soldier must annually pass this physical fitness test, which includes a sprint test (11 × 10-m shuttle test), a flexed-arm hang test and a 1000-m run. The entire test must be completed in 90 min. Minimum test requirements (see Table 1) must be fulfilled regardless of gender or age [11]. See the “Methods” chapter for a detailed description of the individual test items. However, the BFT is not mandatory for the reenlistment of a soldier.

**Table 1:** Minimum pass requirements of BFT

Athletic discipline	Minimum requirements
11x10-m sprint test	≤60 s
Flexed-arm hang	≥5 s
1000-m run	≤390 s

Minimum pass requirements of the Basic Fitness Test [11]

In addition, the German Armed Forces still conduct bicycle ergometer tests as part of their medical examinations for reenlistments, as is customary in other armed forces. Pilots, for example, undergo bicycle ergometer tests, not only to exclude cardiovascular diseases but also to confirm that they meet minimum physical requirements [12, 13]. While ergometer tests can assess physical fitness in a standardized way, regardless of weather conditions, they only provide information about a subject’s treadmill- or bicycle-specific fitness.

Because soldiers need more than maximal aerobic power for the successful performance of their military tasks, and the bicycle ergometer test only assesses this skill, it is important to compare the results of the bicycle ergometer test with the results of the BFT to determine if the physical fitness of soldiers can be assessed using only one test. Therefore, the objective of the present study is 1) to analyze the degree to which BFT results are consistent with the output measured during bicycle ergometer tests and 2) to record the rate of false-positive and false-negative results in the assessment of physical fitness obtained during bicycle ergometer tests compared with the BFT.

## Methods

As part of a retrospective study, health assessments from 323 reenlistment and “survival on operations” examinations conducted at the Augustdorf Major Medical Clinic with Specialty Services, Detmold branch, Internal Medicine clinic were evaluated (from 2010 to 2012) and compared with the results on the BFT. “Survival on operations” examinations were included to increase the overall number of data sets, as they were largely identical to reenlistment examinations and were all conducted on young military personnel. Only men were included in this study because only a small number of women served in the German Army Forces.

On average, the subjects were 24.3 ± 2.6 years old, 179.7 ± 6.8 cm tall and weighed 82.5 ± 11.7 kg (BMI: 25.5 ± 3.2 kg/m^2^). Their average absolute and relative output during the bicycle ergometer test was 239 ± 31 watts and 2.9 ± 0.4 watts, respectively, per kilogram of body weight. The average BFT output was 42.0 ± 3.6 s (11 × 10-m run), 44.3 ± 19.8 s (flexed-arm hang) and 261.8 ± 39.3 s (1000-m run).

The project was given a positive assessment by the Bundeswehr Medical Service, is registered under the research number 01KS-S-631314 and meets the international ethical standards [14].

### Bicycle ergometer test

Ergometer tests conducted on a bicycle ergometer were analyzed for the maximum output achieved and for the relative output per kilogram of body weight. The tests were performed according to a standardized protocol, starting at 100 watts and increasing by 50 watts every 2 min.

### BFT

The BFT data of all soldiers were provided by their companies and correlated with the bicycle ergometer test output. The data were collected during the routine BFT in the same year of the ergometer test.

The BFT comprises three individual physical fitness test items, which must be completed in a fixed sequence [11] and is observed by an instructor. The first test is the 11 × 10-m sprint test. Subjects start lying prone on an exercise mat with their arms crossed behind their backs. After the start signal, they run around a marker at a 10-m distance and return to the mat. Once subjects return to the mat, they must lie down again and clap their hands behind their backs. This process is repeated five times. After lying down for the fifth time, subjects stand up for a final run to the 10-m marker, where they complete the 11 × 10-m sprint test. Time was measured to one decimal place.

During the flexed-arm hang test, the subjects must keep their chin above a pull-up bar with their arms flexed and hold their body weight in a static position with their hips and knees extended. The hands should be placed on the bar shoulder-width apart. Timing starts when the start signal is given and ends when the chin drops below the bar.

The 1000-m run is completed in a group on a 400-m running track. Timing starts with the start signal and ends when the subjects cross the 1000-m marker.

The overall score is calculated based on the absolute measurements of every discipline in seconds. These are converted into points with 100 points equaling the minimum requirement with the following set phrase:

- Points 11 × 10-m sprint run = 1100–(16.667 × time sprint test [s]);
- Points flexed-arm hang test = 75 + (5 × time flexed-arm hang test [s]); and
- Points 1000-m run = 100 + ((390 – time 1000-m run [s]) × 1.81818181).
- All three disciplines are weighted equally in the calculation of the overall score.

### Data collection and statistics

The data were stored in a Microsoft Access 2010® database in pseudonymised form and evaluated using IBM SPSS Statistics 22. In addition to descriptive statistics (mean value, standard deviation), a fourfold contingency table was used to calculate false-positive and false-negative results; a Pearson’s correlation was conducted, and a linear regression analysis was run. Differences between groups were analyzed using an independent samples *t*-test. A *P*-value lower than 0.05 was considered statistically significant.

## Results

Out of 323 soldiers, 310 soldiers passed both the bicycle ergometer test and the BFT. Two soldiers were evaluated as physically fit during the bicycle ergometer test but did not pass the BFT. Eleven soldiers were not able to meet the minimum requirements of the bicycle ergometer test but passed the BFT. In 96.0 % of all cases, the subjects passed both the bicycle ergometer test and the BFT (Table 2).

**Table 2:** Fourfold contingency table of the results of BFT and ergometry

	Failed BFT	Passed BFT
Failed cardiac stress	0	**11**
Passed cardiac stress test	**2**	310

Fourfold contingency table; false positive and false negative results are highlighted in bold letters

For the endurance test only, a good correlation was shown between the bicycle ergometer test results and the results achieved in the BFT disciplines (*r* = −0.237, *P* < 0.001). The 11 × 10-m sprint test and the flexed-arm hang showed no correlation with the absolute output (*r* = −0.068, *r* = 0.009, respectively) and were considered statistically insignificant with *P*-values > 0.05 (Table 3). The correlation of the overall BFT score with the absolute output measured during the bicycle ergometer test was significant (*r* = 0.132, *P* = 0.018).

**Table 3:** Correlation between BFT and absolute output on bicycle stress test

Athletic discipline	*r*	*P*	*R* ^2^
11 × 10-m sprint test [s]	−0.068	0.224	0.005
Flexed-arm hang [s]	0.009	0.873	0.000
1000-m run [s]	−0.237	**<0.001**	0.056
Total output [score]	0.132	**0.018**	0.017

Correlation between the measured BFT output/score and the absolute output measured during the bicycle stress test including regression analysis variance; significance results are highlithted in bold; *n* = 323

**Fig. 1:**
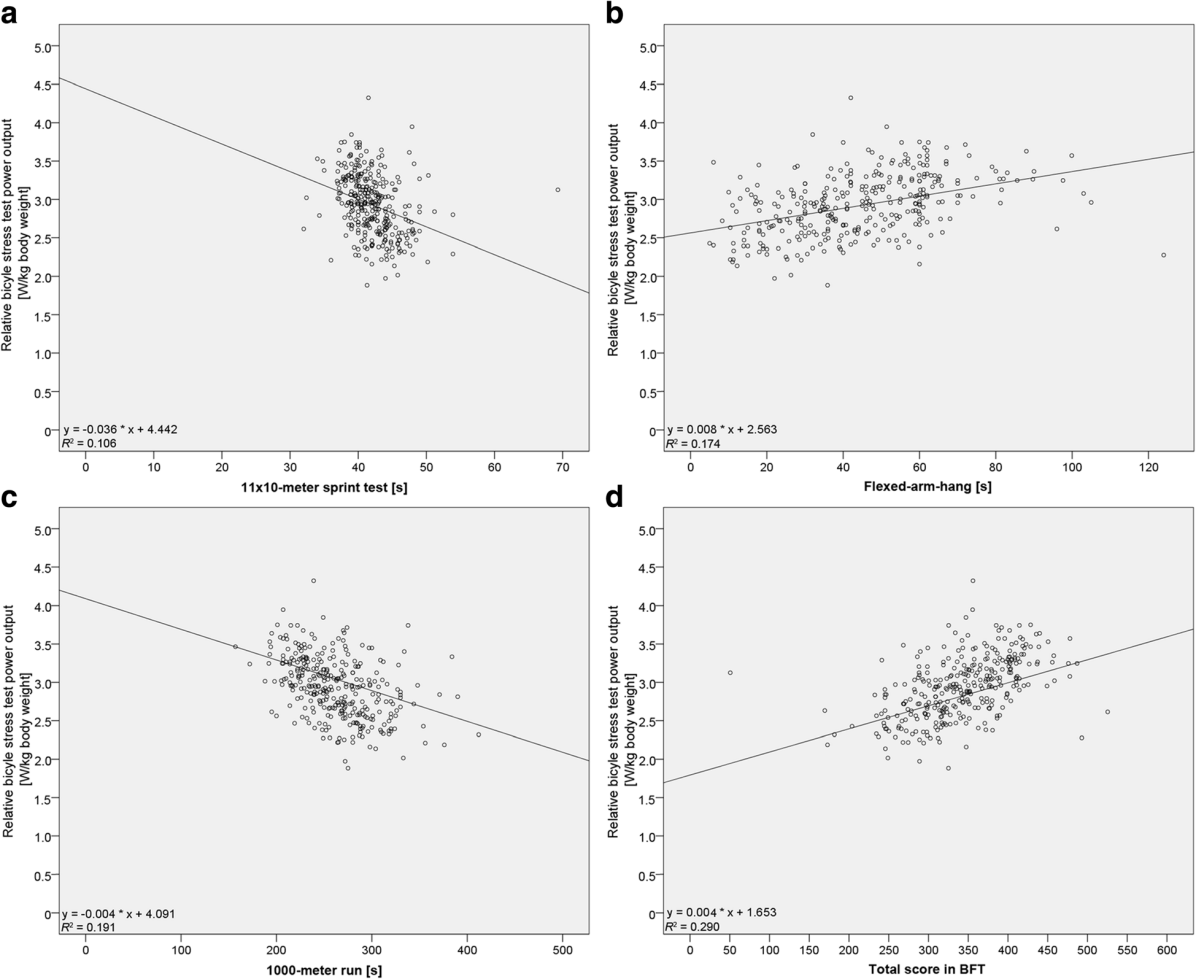
Scatter plots of the correlations. Scatter plots of the results achieved in the sprint test (**a**), flexed-arm hang (**b**) 1000-m run (**c**) and total score of BFT (**d**) in correlation with the relative power output measured during the bicycle stress test and illustration of the relevant regression lines. *n* = 323

Based on the correlations of the BFT results in the three disciplines and the overall BFT score with the output measured during the bicycle ergometer test in relation to body weight, we conclude that with regard to all individual disciplines, as well as to the BFT score, the correlations were highly significant (11 × 10-m sprint test *r* = −0.325, flexed-arm hang *r* = 0.417 and 1000-m run *r* = −0.437, BFT in total *r* = 0.538, all *P* < 0.001, Table 4). In Fig. 1, this is illustrated by scatter plots with regression lines.

**Table 4:** Correlation between BFT and relative output on bicycle stress test

Athletic discipline	*r*	*P*	*R* ^2^
11x10-m sprint test [s]	−0.325	**<0.001**	0.106
Flexed-arm hang [s]	0.417	**<0.001**	0.174
1000-m run [s]	−0.437	**<0.001**	0.191
Total output [score]	0.538	**<0.001**	0.290

Correlation between the measured BFT output/score and the relative power output (watts/kg) measured during the bicycle stress test including regression analysis variance; significance results are highlithted in bold; *n =* 323

## Discussion

The results presented here demonstrate, based on a large sample, a significant correlation between the individual disciplines of the BFT (11 × 10-m sprint test, flexed-arm hang and 1000-m run) and the output measured during a bicycle ergometer test completed by the examined cohort of young subjects. The results also show that the BFT provides a similar assessment of physical fitness. The 4.0 % false-negative and false-positive rate, however, showed that the bicycle ergometer test, in contrast with the BFT, which was designed to test more than only maximal aerobic power, resulted in an underestimation of the level of physical fitness and fitness for service. In addition to the size of the sample, the diversity of the areas of physical fitness examined is a distinguishing feature of the present study.

Bicycle ergometer tests are controversial as part of assessments [15, 16]. The reliability of these tests is rated as low in regard to excluding cardiac arrhythmia and detecting coronary heart diseases in a young and healthy cohort [17, 18]. Bicycle ergometer tests as part of medical check-ups for athletes are therefore not recommended under the age of 35 or 40 [19–24]. It is also widely accepted that bicycle ergometer tests are only suitable to evaluate bicycle-specific fitness [16]. Moreover, subjects with a higher body weight have the advantage that the impact of their additional weight is reduced in this evaluation [25]. Ergometer tests have advantages over separate physical fitness tests. They can be performed in a standardized way, independent of weather conditions, and require little space. It should be noted, however, that they require more personnel.

The BFT presented here requires little effort because of its conditions. It merely requires a suitable gym for the sprint and the flexed-arm hang tests, as well as the necessary materials (mats, cones to mark the floor, pull-up bar) and a track suitable for running a 1000-m distance (e.g., a 400-m track). The high correlation of the individual BFT disciplines and the overall BFT score with the bicycle ergometer test (both in absolute values and relative to body mass) indicates that the BFT can be used as an alternative to bicycle ergometer tests. Because it targets the physical fitness skills of endurance, strength and speed, the BFT is the more suitable test procedure.

The high correlation between the individual BFT items and the bicycle ergometer test was confirmed for other tests as well. Williford et al. [13] analyzed the correlation between bicycle ergometer tests and maximal treadmill tests and found a correlation of *r* = 0.74. The maximal oxygen uptake (VO_2max_), however, was considerably lower during the bicycle ergometer test (−17 %). Basset et al. [26] also found comparability between bicycle and treadmill ergometer tests when examining 6 triathletes, 6 runners and 6 cyclists. They came to the conclusion that in both tests the heart rate and the percentage of the VO_2max_ were comparable. Jaskólska et al. [27] observed a high correlation (*r* = 0.71–0.86) between these two types of ergometer tests as well in their examination of 32 male subjects. Carey et al. [28] did not identify any differences in the maximum heart rate and the VO_2max_ in the examination of 16 experienced triathletes, they did detect significant differences regarding the determination of the anaerobic threshold. Although we can generally assume a relatively good correlation between both test systems, we must take into consideration that both tests were conducted as stationary laboratory tests and that the majority of examined subjects were athletes who were well-trained in the relevant athletic disciplines.

In a study by Grant et al. [29], a very high correlation (*r* = 0.92) was observed in 22 young male subjects between the 12-min Cooper test and submaximal cycling output. The results of our study are consistent in this respect because we also observed a very high correlation between the 1000-m run and the bicycle ergometer test output.

The study of Grant et al. [29] and a study of Cairney et al. [30] note a high correlation between shuttle runs and bicycle ergometer tests. Grant et al. conducted a multi-stage progressive shuttle run test and detected a correlation of *r* = 0.86 with the bicycle ergometer test while Cairney et al. examined children doing a 20-m shuttle run and found a correlation of *r* = 0.71. These results are thus also consistent with the findings of the present study although the test structure referred to in the References section differs from the shuttle run (11 × 10-m sprint test) examined by us.

Whereas subjects with a higher body weight have an advantage in bicycle ergometer tests [25], a review carried out by Vanderburgh [31] showed that in the fitness tests common in the US Army, Air Force and Navy, subjects with lower body weight were able to perform better. The present study also demonstrates that weight has an influence on the results achieved during the flexed-arm hang. Only the relative output measured during the bicycle ergometer test correlated with the flexed-arm hang output. In addition, regression analysis variance increased for all test items when the output in the BFT disciplines was compared with the relative output measured during the bicycle ergometer test. This is not surprising, as subjects with a higher body weight achieve considerably lower results in the flexed-arm hang test in particular, whereas in the bicycle ergometer test their results are higher in comparison.

This study does have some limitations. Because the data analysis was retrospective, ergometer test and BFT data were compared irrespective of how much time had passed between the tests. We can therefore not rule out that the physical fitness of the subjects had improved or worsened significantly during this period. Among other things, this could explain the number of false-negative and false-positive results. To provide reliable information on the correlation between the bicycle ergometer test and BFT output, a prospective randomized study with short intervals between the two tests should be conducted. This, however, was beyond the scope of this study.

Because the overall proportion of women in the German armed forces is low (approx. 10 %), only men were included in this study. Therefore, these results cannot be generalized to physical fitness examinations of women. Moreover, it is possible that the group of subjects on which the study is based is not representative of the respective locations. It is conceivable, for example, that only particularly unathletic or sick persons or, on the contrary, especially fit or healthy persons presented to the specialist clinic. This can be considered unlikely because many different locations and unit physicians have referred personnel to the Specialist Clinic for Internal Medicine for medical examination, and because an interim evaluation of the cohort of temporary career volunteers used for the analysis (comprising the assessments of the period from 2007 to 2010) includes both soldiers with a high level of physical fitness and a considerable number of soldiers that were unfit for service [32]. It can therefore be assumed that the overall sample of 323 soldiers has not been affected by significant selection bias through the referral/presentation of subjects.

## Conclusions

It can be concluded that the BFT results measured in three physical fitness test items have a high correlation with the output measured during bicycle ergometer tests, that the rate of false-positive and false-negative results is low and that the test items therefore represent an appropriate measuring instrument because they require few equipment and less time, and a large number of subjects can be assessed. We suggest that it would be more useful to assess the physical fitness of this group of subjects exclusively on the basis of the BFT instead of using the bicycle ergometer test.
